# Bone Disease in Thalassemia: A Frequent and Still Unresolved Problem

**DOI:** 10.1359/jbmr.080505

**Published:** 2008-05-19

**Authors:** Maria G Vogiatzi, Eric A Macklin, Ellen B Fung, Angela M Cheung, Elliot Vichinsky, Nancy Olivieri, Melanie Kirby, Janet L Kwiatkowski, Melody Cunningham, Ingrid A Holm, Joseph Lane, Robert Schneider, Martin Fleisher, Robert W Grady, Charles C Peterson, Patricia J Giardina

**Affiliations:** 1 Department of Pediatrics, Weill Medical College of CornellNew York, New York, USA; 2 New England Research InstitutesWatertown, Massachusetts, USA; 3 Children's Hospital OaklandOakland, California, USA; 4 Department of Medicine, University Health Network and Mount Sinai HospitalToronto, Canada; 5 Toronto Hospital for Sick ChildrenToronto, Canada; 6 Division of Hematology, Children's Hospital of Philadelphia and Department of Pediatrics, University of Pennsylvania School of MedicinePhiladelphia, Pennsylvania, USA; 7 Division of Hematology & Oncology, Children's Hospital BostonBoston, Massachusetts, USA; 8 Hospital for Special SurgeryNew York, New York, USA; 9 Memorial Sloan-Kettering Cancer CancerNew York, New York, USA; 10 National Heart, Lung, and Blood InstituteNIH, Bethesda, Maryland, USA

**Keywords:** DXA, BMD, fractures, vertebral morphometry, thalassemia

## Abstract

Adults with β thalassemia major frequently have low BMD, fractures, and bone pain. The purpose of this study was to determine the prevalence of low BMD, fractures, and bone pain in all thalassemia syndromes in childhood, adolescence, and adulthood, associations of BMD with fractures and bone pain, and etiology of bone disease in thalassemia. Patients of all thalassemia syndromes in the Thalassemia Clinical Research Network, ≥6 yr of age, with no preexisting medical condition affecting bone mass or requiring steroids, participated. We measured spine and femur BMD and whole body BMC by DXA and assessed vertebral abnormalities by morphometric X-ray absorptiometry (MXA). Medical history by interview and review of medical records, physical examinations, and blood and urine collections were performed. Three hundred sixty-one subjects, 49% male, with a mean age of 23.2 yr (range, 6.1–75 yr), were studied. Spine and femur BMD Z-scores < −2 occurred in 46% and 25% of participants, respectively. Greater age, lower weight, hypogonadism, and increased bone turnover were strong independent predictors of low bone mass regardless of thalassemia syndrome. Peak bone mass was suboptimal. Thirty-six percent of patients had a history of fractures, and 34% reported bone pain. BMD was negatively associated with fractures but not with bone pain. Nine percent of participants had uniformly decreased height of several vertebrae by MXA, which was associated with the use of iron chelator deferoxamine before 6 yr of age. In patients with thalassemia, low BMD and fractures occur frequently and independently of the particular syndrome. Peak bone mass is suboptimal. Low BMD is associated with hypogonadism, increased bone turnover, and an increased risk for fractures.

## INTRODUCTION

The thalassemia syndromes are a group of congenital hemolytic anemias characterized by the reduced or absent synthesis of one or more globin chains of hemoglobin.(1) The two most common forms are the α and β thalassemias. β Thalassemias include β thalassemia major (β TM), β thalassemia intermedia (β TI), and E-β thalassemia (E-β), and result from >200 mutations affecting the β globin gene cluster. The α thalassemia syndromes include hemoglobin H disease (HbH, or three gene α thalassemia), the co-inheritance of HbH disease with hemoglobin Constant Spring (HbH/CS) or other nondeletional α-globin mutations, and homozygous α thalassemia (Hz α).

The hallmark of thalassemia is an imbalance in α to β globin production, with the severity of anemia related to the degree of this imbalance. The variable phenotypic expression within and among the thalassemia syndromes results in a wide spectrum of anemia, which may require periodic or regular transfusions to sustain life.(1) Typically, patients with β TM, Hz α, and HbH/CS and some with E-β require regular transfusions, whereas β TI and HbH are transfused sporadically, if at all. Because there is no physiologic means of excreting iron, transfusion therapy leads to progressive iron overload and ultimately multiple endocrinopathies, and potentially, lethal hepatic and/or cardiac complications. Iron chelation therapy with deferoxamine (DFO), introduced in the mid-1960s, has changed the course of transfusional iron overload, reducing iron excess and dramatically prolonging survival.(2,3)

Historically, β TM was associated with marked osseous changes, and in particular, facial and limb deformities that were attributed to bone marrow expansion and cortical thinning caused by massive ineffective erythropoiesis.(4,5) The introduction of regular transfusion therapy in the mid-1960s to maintain a near-normal hemoglobin level resulted in diminution or prevention of bone deformities.(6) Therefore, the detection of low bone mass in many regularly transfused and well-chelated β TM patients over the last decade was quite unexpected.(7,8) There is now a growing awareness that many transfusion-dependent adult patients with β TM and β TI suffer from long-standing bone pain and low bone mass.(9,10) The presence of low bone mass, fractures, and bone pain (referred to collectively in this paper as bone disease) in patients with other thalassemia syndromes is unclear. Little is known about the prevalence of low bone mass in children and adolescents with thalassemia or the current rate of fractures and associations with bone mass and bone pain. Furthermore, the etiology of bone disease in thalassemia is poorly understood. A number of studies have examined the effect of various conditions on the pathogenesis of bone disease, including ineffective erythropoiesis, iron overload, treatment with DFO, vitamin D concentrations, the influence of endocrinopathies, such as hypogonadism and growth hormone deficiency, and the thalassemia genotype.(11–17) The results have frequently been contradictory, and the studies have been hindered by small sample size and variable practice regimens.

The Thalassemia Clinical Research Network (TCRN) consists of five thalassemia centers in North America and their associated satellite sites with access to patients having both α and β thalassemia syndromes. The TCRN performed a cross-sectional study of BMD, fracture, and bone pain histories and vertebral morphology among its patients to better understand the severity of bone disease in thalassemia and its etiology. The purpose of the study was to determine the prevalence of low bone mass, fractures, and bone pain across all ages and thalassemia syndromes and to describe associations between bone mass and fractures. We evaluated the associations of anemia, transfusion and chelation management, iron burden and related endocrinopathies, and environmental and genetic factors with bone mass, fractures, and pain. We also sought to identify changes in bone turnover that are involved in the development of bone disease in thalassemia. From these results, we hope to develop future preventative strategies and more effective treatment regimens.

## MATERIALS AND METHODS

### Study protocol

TCRN patients of all thalassemia genotypes, ≥6 yr of age, were eligible for this study. Exclusion criteria included pregnancy, any known preexisting medical condition known to affect bone mass, and chronic systemic administration of steroids. The protocol was approved by the TCRN Data and Safety Monitoring Board and by the ethical review boards of all TCRN institutions. All participants provided signed informed consent.

Study participants underwent a complete physical examination and measurement of BMD and vertebral morphometry by DXA. A medical history was obtained by interview and review of medical records. Dietary calcium intake was estimated from a 46-item, self-completed food frequency questionnaire. Physical activity was reported as very light, light, moderate, active, or very active using a standardized questionnaire. A fasting morning blood sample was obtained for measurement of N-telopeptides (NTX), C-telopeptides (CTX), bone-specific alkaline phosphatase (BSALP), free thyroxine and thyroid-stimulating hormone (TSH), IGF1, IGF binding protein 3 (IGFBP3), serum gonadotropins (luteinizing hormone [LH] and follicle-stimulating hormone [FSH]), testosterone (males), 25-hydroxyvitamin D (25 vit D), 1,25-dihydroxyvitamin D (1,25 vit D), intact PTH, serum calcium, phosphorus, ferritin, and transferrin receptor concentrations. A second morning void was collected for measurement of urinary NTX and deoxypyridinoline cross-links (D-Pyr). A 24-h urine collection was performed for measurement of calcium excretion. Bone age was determined from X-rays of the left hand and wrist of participants <20 yr of age and read locally according to the method of Greulich and Pyle.(18)

### Procedures

#### BMD and morphometry

BMD of the lumbar spine (L_1_–L_4_; spine) and left proximal femur (femur), a lateral lumbar scan (lat spine) for calculation of volumetric lumbar BMD, and whole body BMC were measured by Hologic QDR 4500 scanners with Delphi software upgrades. The precision of spine and femur BMD measurements was determined by duplicate measurement of 140 and 138 subjects, respectively. The root mean square errors (RMSEs) of spine and femur BMD were 0.022 (CV = 4.6%) and 0.009 g/cm^2^ (CV = 1.3%), respectively. Except for assessing precision, only the first of the two duplicate scans was used for statistical analysis. The unanalyzed BMD images were processed and analyzed by a single, blinded technologist and radiologist at a central processing center (Hospital for Special Surgery, New York, NY, USA). BMD results were analyzed at a distinct Data Coordinating Center (DCC).

The presence of vertebral fractures and deformities was assessed by morphometric X-ray absorptiometry (MXA) using Hologic Instant Vertebral Assessment (IVA). Findings from T_10_ to L_4_ were analyzed by a single senior radiologist (RS). The severity of deformities was graded using the Genant method(19): grade I, 20–25% reduction in anterior, posterior, or midvertebral height; grade II, 25–40% reduction; grade III, >40% reduction.

#### Laboratory assays

Urine and serum samples from each participant were stored at –80°C and analyzed as a batch at a central facility. Serum NTX and CTX and urine NTX were measured by a competitive inhibition, enzyme-linked immunoabsorbent assay, D-Pyr by a solid-phase, enzyme-labeled chemiluminescent immunometric assay, IGF1, IGFBP3, and BSALP by a solid-phase, enzyme-linked immunoassay (ELISA), 25 vit D by competitive radioimmunoassay after extraction, 1,25 vit D by column chromatography and radioimmunoassay, intact PTH by immunochemiluminometric assay, and free T4, LH, FSH, and TSH by high sensitivity heterogeneous sandwich separation assay. Testosterone was measured by a solid-phase, competitive radioimmunoassay. Serum ferritin levels were determined by a radioimmunoassay (RIA, T-14; Ramco Laboratories, Houston, TX, USA). Serum transferrin receptor concentrations were measured by an enzyme immunoassay (EIA; T-94; Ramco Laboratories).

### Statistical analysis

#### Calculated variables

Patients with β thalassemia were classified as having TM if they had received eight or more transfusions during the 12 mo before entering the study or as TI if less.

Body mass index (BMI) was calculated as kilograms per meters squared. Anthropometric Z-scores were calculated relative to age- and sex-specific norms for whites produced by the CDC from NHANES III data. Midparental heights were calculated as the average parental height ± 6.5 cm for boys and girls, respectively. BMD Z-scores were calculated relative to age-, sex-, and race-specific norms provided by Hologic. Asian and Middle Eastern individuals were compared with white norms. Bone age-adjusted BMD Z-scores were calculated for participants <20 yr old using each participant's estimated bone age for selecting the appropriate norm.

Right skewed blood and urine assay results were log-transformed to reduce the influence of highly leveraged points to better conform to the distributional assumptions of the models applied. Vitamin D deficiency was defined as a 25 vit D concentration <11 ng/ml and insufficiency as a concentration between 11 and 30 ng/ml. The presence of hypogonadism, growth hormone deficiency, hypothyroidism, hypoparathyroidism, or diabetes mellitus was defined as having an identified clinical history or prescribed treatment. In addition, hypoparathyroidism was defined as an intact PTH level below normal range for our assay in the presence of albumin-adjusted hypocalcemia (serum calcium < 8.5 mg/dl). Among females, hypogonadism was defined as lack of spontaneous menses after the age of 16, having a history of using hormone replacement therapy (HRT) for failure to proceed through puberty or loss of menses before age 40, or current use of HRT. Hypogonadism in males was determined by the prescription of HRT (testosterone or HCG) or by having a serum testosterone concentration lower than established norms for age. Inadequate gonadal steroid replacement in hypogonadal males was confirmed by having a serum testosterone concentration lower than established norms for age despite prescribed HRT. Fasting hyperglycemia (blood glucose > 126 mg/dl), having a history of fasting hyperglycemia or of an abnormal glucose response to oral glucose tolerance test (blood glucose > 126 mg/dl at 0 min or >200 mg/dl at 120 min), having a history of prescribed therapy with oral hypoglycemics or insulin, or current therapy with oral hypoglycemics or insulin was used to determine the presence of diabetes. The diagnosis of growth hormone deficiency in children with growth failure was made after endocrine referral at each participating site and appropriate testing.

Self-reported bone and joint pain during the 30 days before the study interview was classified as an ordinal pain severity index according to the participant's use of pain medications as follows: no reported pain, reported pain not requiring use of medications, pain treated with over-the-counter medications, and pain treated with prescription pain killers.

#### Analyses

Continuous variables were summarized as means, SDs, and ranges. Categorical variables were summarized as simple percentages. Analyses of DXA results and bone turnover markers were analyzed in general linear models controlling for age as a covariate using partial-linear splines with either one node at age 20 yr for DXA results or two nodes at ages 11 and 20 yr for bone turnover markers. These intervals are the outer range of initiation and completion of puberty across both sexes and matched breakpoints in the distribution of the data. In addition to age, analyses of whole body BMC included sex and race (Asian, white, and other race) as covariates in all models. Pairwise comparisons among least-square means of categorical predictors were adjusted for multiple comparisons by the Tukey-Kramer method. Ages of peak bone mass were estimated by locally linear generalized additive models,(20) with bias-corrected and accelerated bootstrap CIs.(21) Age-dependent incidence of fractures was analyzed in proportional means models,(22) with age at study entry included as a covariate in all models. Pain severity was analyzed by ordinal logistic regression, with age and sex included as covariates. Presence of vertebral fractures and deformities identified by MXA were analyzed by binary logistic regression with age as a covariate. Wald *p* values and profile likelihood CIs are reported from proportional means and logistic regression models. Multiple regression models were selected from all analysis variables by stepwise regression with final variable selection by the Bayesian information criterion.

Analyses were generally exploratory, with the aim of describing observed patterns in the data. Multiple comparison corrections for the large number of models and outcome measures analyzed were not made. All inferences were based on two-tailed tests with a threshold of α = 0.05 for declaring significance. Analyses were conducted using SAS (version 9.1.3; SAS Institute, Cary, NC, USA) and S-Plus (version 7.0; Insightful, Seattle, WA, USA).

## RESULTS

### Participant characteristics

A total of 386 individuals consented to the study, and 372 completed the protocol, of whom 9 were excluded because of prior stem cell transplantation, chronic use of systemic steroids, or incomplete data. In addition, only two participants had Hz α and were not included in our analysis because their sample size precluded meaningful interpretation of their data.

Characteristics of the 361 eligible participants who completed DXA scanning and had β, E-β, or common α thalassemias are presented in Table 1. Mean height and weight were within 2 SDs of the general population mean for all thalassemia syndromes. However, 95 participants (27% of 352 with height measurements) had significant growth failure, with height Z-scores below −2 SD. The mean bone age of children and adolescents was close to their chronological age. Hypogonadism was the most frequent endocrinopathy, affecting 34% of females and 38% of males. Vitamin D deficiency (25 vit D < 11 ng/ml) and insufficiency (25 vit D = 11–30 ng/ml) were found in 12% and 70% of participants, respectively. Growth hormone deficiency was present in 9.6%, diabetes mellitus in 9.2%, hypothyroidism in 8.7%, and hypoparathyroidism in 1.4% of study participants. Twenty-nine adults were treated with a bisphosphonate.

**Table 1:** Characteristics of Study Participants^*^

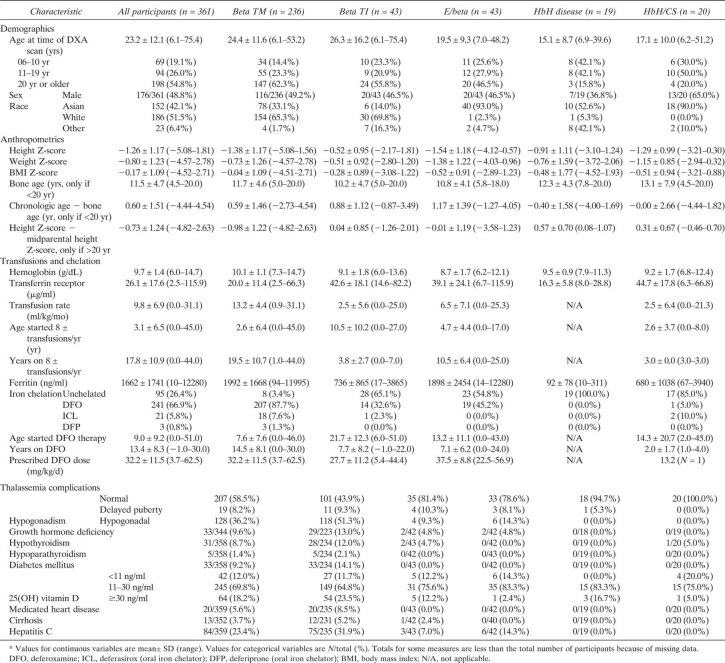

### Bone mass

#### Demographic profile

**FIG. 1:**
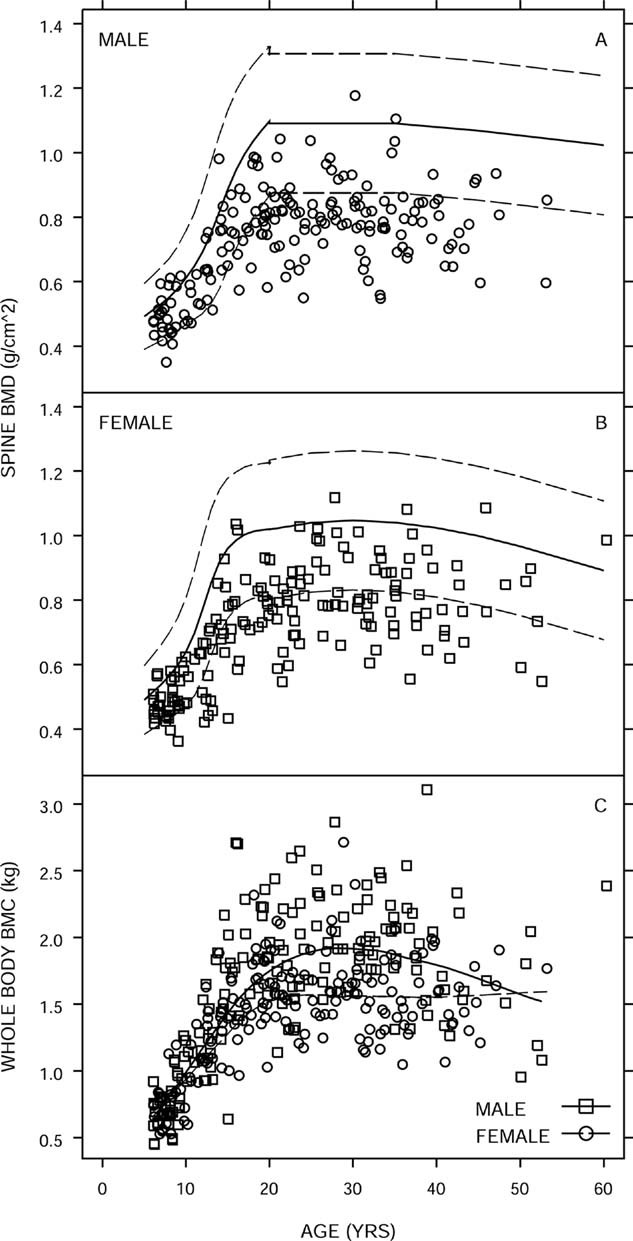
Bone mass vs. age. (A and B) Spine BMD (g/cm^2^) with age-dependent reference norms for whites (solid line) and ±2 SD (dashed lines) for males (A) and females (B). (C) Whole body BMC (kg) with locally weighted regressions for males (solid line) and females (dashed line). One individual age 75 yr is omitted (symbols: male, □; female, ○).

**FIG. 2:**
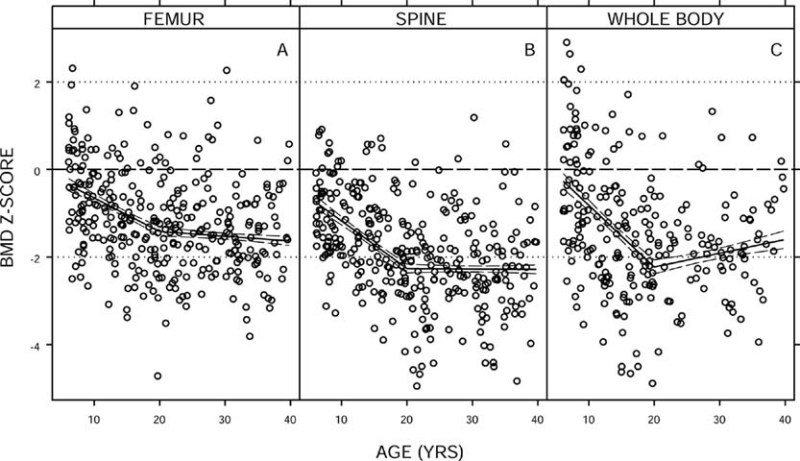
Spine, femur, and total body BMD Z-scores with a partial-linear spline (solid line) and 95% CI (dashed lines). Individuals >40 yr of age are omitted.

Spine, femur, and whole body BMD were close to age norms at age 6 yr. A strong negative association between bone mass and age, which was more marked in the age group 11–19 years of age was observed (−0.84 SD/5 yr; 95% CI, −1.10 to −0.59 SD; *p* < 0.001 for whole body BMD among participants < 20 yr of age; Figs. 1 and 2). The rates of spine BMD Z-scores less than –2 increased from 8.7% among participants 6–10 yr old to 44% in those 11–19 yr old and 61% in patients >20 yr old. Above age 20 yr, the association between spine BMD and age was nearly constant relative to age norms (−0.008 SD/5 yr; 95% CI, −0.08–0.07 SD; *p* = 0.25; Figs. 1 and 2). Similar patterns of BMD Z-scores versus age were observed for the femur. Sex or race did not account for the differences in the spine and femur BMD Z-scores. Norms for adult male whole body BMD were not available, but among females, whole body BMD Z-scores increased modestly but insignificantly (*p* = 0.15) above age 20 yr, perhaps reflecting a survival bias. Peak bone mass, estimated as the age with maximum whole body BMC, occurred at 22.4 (95% CI, 19.0–31.8) and 29.8 yr (95% CI, 23.5–34.0) among females and males, respectively (Fig. 1C). Among participants 20–29 yr of age, 86% (75/87) had spine BMD Z-scores less than −1 SD, indicating suboptimal peak bone mass.

#### Thalassemia syndromes

**FIG. 3:**
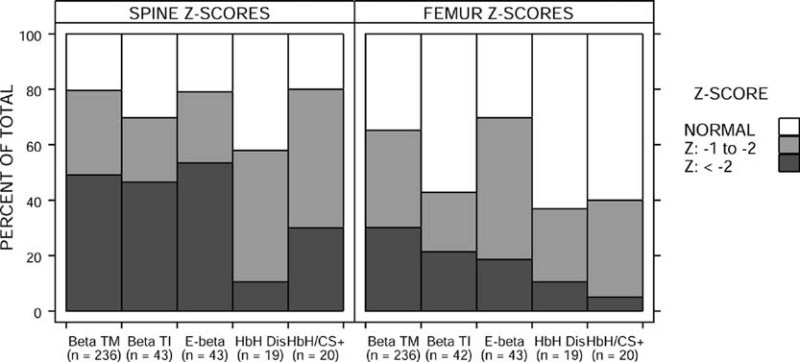
Prevalence of low (Z-score < −2 SD), reduced (−2 SD ≤ Z-score < −1 SD), and normal (Z-score ≥ −1 SD) spine and femur Z-scores by thalassemia syndrome.

Low spine and femur BMD (Table 2; Fig. 3) and whole body BMD were prevalent among all thalassemia syndromes. Frequencies of BMD Z-scores below −1 SD ranged from 58% to 80% for spine and from 37% to 70% for femur. Whole body BMD normative data were available only for children and adolescents and adult females. Among children and adolescents, prevalence of whole body BMD Z-scores below –1 SD was similarly high across all thalassemia syndromes, ranging from 50% to 74%.

**Table 2:** Bone Disease Among Thalassemia Syndromes^*^

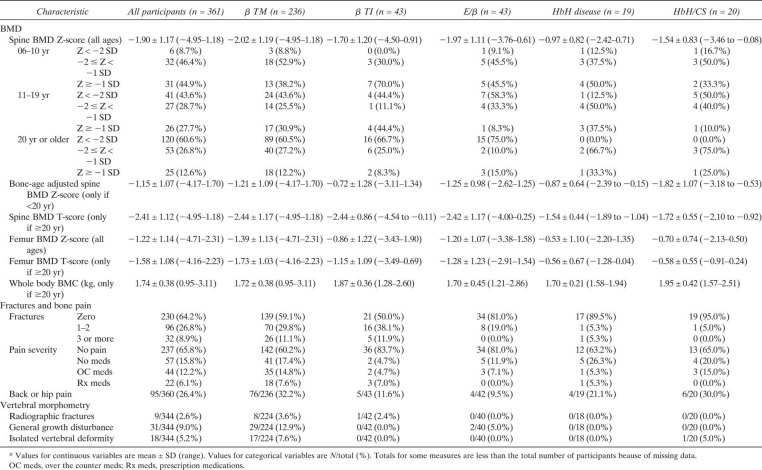

Estimated mean spine BMD Z-scores of a 20 yr old ranged from −1.64 to −2.37 SD (mean [95% CI]: β TM, −2.37 [−2.64,−2.11]; β TI, −2.15 [−2.56,−1.75]; E-β, −2.34 [−2.74,−1.95]; HbH Dis, −1.64 [−2.16,−1.11]; HbH/CS, −2.00 [−2.52,−1.49]), controlling for sex and race. Age-, sex-, and race-adjusted BMD Z-scores and whole body BMC were lower among β TM and E-β participants and higher among β TI, HbH disease, and HbH/CS participants (spine, *p* = 0.062; femur, *p* = 0.028; whole body BMC, *p* = 0.025; Fig. 3), although individual pairwise comparisons were not significant after controlling for multiple comparisons. Spine BMD Z-scores of β TM and TI participants did not differ significantly (*p* = 0.74).

Spine BMD was lower than femur BMD relative to their norms (0.67 SD; 95% CI, 0.59–0.76 SD; *p* < 0.001). The difference between spine and femur BMD Z-scores increased significantly across adolescence (*p* < 0.001) and was greater among males than females (0.25 SD; 95% CI, 0.08–0.42 SD; *p* = 0.004) but was not associated with race or thalassemia syndrome.

#### Associations

Results of analyses of spine and femur BMD Z-scores controlling for age, and whole body BMC controlling for age, sex, and race are presented in Table 3. These include associations with demographic, genetic, anthropometric, and environmental factors, transfusion and chelation parameters, thalassemia-related endocrinopathies and co-morbidities, markers of bone turnover, serum IGF1 and IGFBP3 concentrations, serum chemistry including Ca and P, and 25 vit D concentrations. A number of positive associations were observed (Table 3). Although hypogonadism was negatively associated with BMD (Table 3), hypogonadal participants who were on gonadal steroid replacement had the same BMD as untreated hypogondal subjects. Participants with vitamin D deficiency had significantly lower BMD (Table 3). The relationship between 25 vit D and BMD Z-score (adjusted for age) was not linear, reaching a plateau at 25 vit D concentrations >15 ng/ml. Finally, no analysis identified any association between treatment with bisphosphonates and bone mass.

**Table 3:** Results of Univariate Analyses of DXA BMD Data^*^

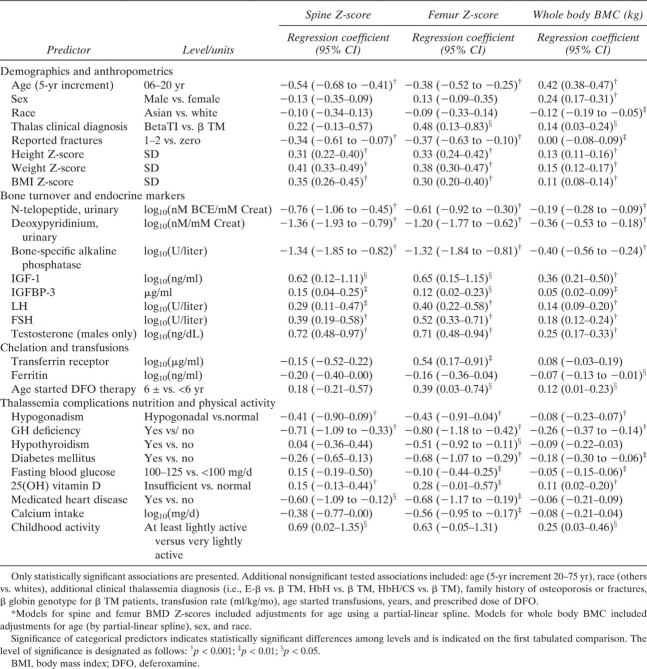

#### Multiple regression analysis

Many of the important predictors of bone mass identified above covary. In multiple regression models, age, weight Z-score, hypogonadism, and markers of bone turnover were consistently strong and independent predictors of spine and femur BMD Z-scores and whole body BMC (Table 4). Spine and femur Z-scores had a strong negative association with age during adolescence, were higher in heavier participants, and were lower in hypogonadal participants and those with elevated urinary D-Pyr (*p* < 0.001 for all). Whole body BMC results were similar except that BSALP was a stronger independent predictor than D-Pyr. In addition to these parameters, 25 vit D was an independent positive predictor of spine BMD Z-scores; family history of osteoporosis was a negative predictor of femur BMD, whereas serum FSH concentrations were a positive predictor of both femur and whole body BMC (Table 4).

**Table 4:** Results of Multivariate Analyses of Spine BMD Z-Scores, Femur BMD Z-Scores, Whole Body BMC, Fracture Rate, Pain Severity, and Vertebral Growth Disturbance

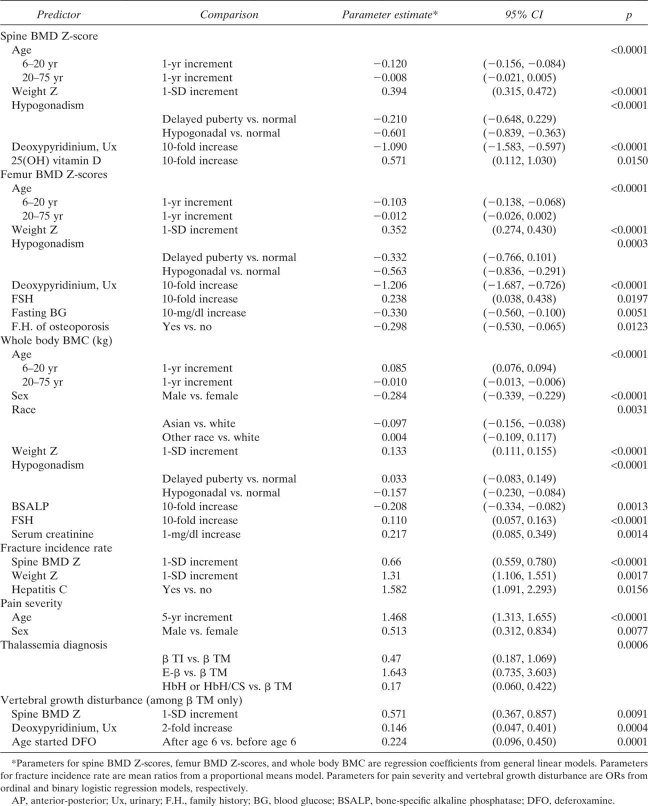

Further analysis focused on participants <20 yr of age to better understand the factors that may affect BMD during adolescence. Among participants <20 yr old, age, weight Z-score, and bone turnover rate continued to be strong, independent predictors of bone mass, but hypogonadism, family history of osteoporosis, and 25 vit D were no longer significant. Sex, race, BSALP, and FSH remained significant, independent predictors of whole body BMC.

#### Calendar age versus bone age

Spine BMD Z-scores in children and adolescents were also analyzed after calculating a bone age-adjusted Z-score (BA Z-score) to control for slow maturation of participants with growth-associated endocrinopathies. This minimally reduced the percentage of children and adolescents with spine and femur BMD Z-scores below −1 SD from 65% to 59% and from 48% to 42%, respectively. Bone age-adjusted spine Z-scores continued to be strongly associated with anthropometric parameters, but significant associations found in this age group between hypogonadism, growth hormone deficiency, IGF-1 levels, and bone turnover markers and calendar age spine BMD Z-scores were not found with bone age-adjusted spine BMD Z-scores.

#### Volumetric BMD

Areal spine BMD using an anterior-posterior (AP) projection may underestimate true BMD in small and growing individuals. Volumetric spine BMD, calculated by combining AP and lateral lumbar projections, may provide a more accurate assessment of BMD in such individuals. Because norms for volumetric BMD were only available for adult women, we studied possible bias in AP estimates by testing for height dependence in the relationship between AP and volumetric BMD. For a given AP BMD, volumetric BMD is greater when height Z-scores are lower (*p* = 0.001); however, the magnitude of difference is small (0.005 g/cm^3^/SD; 95% CI, 0.002–0.008 g/cm^3^/SD) and does not suggest an important effect of size on our findings.

### Bone turnover markers

**FIG. 4:**
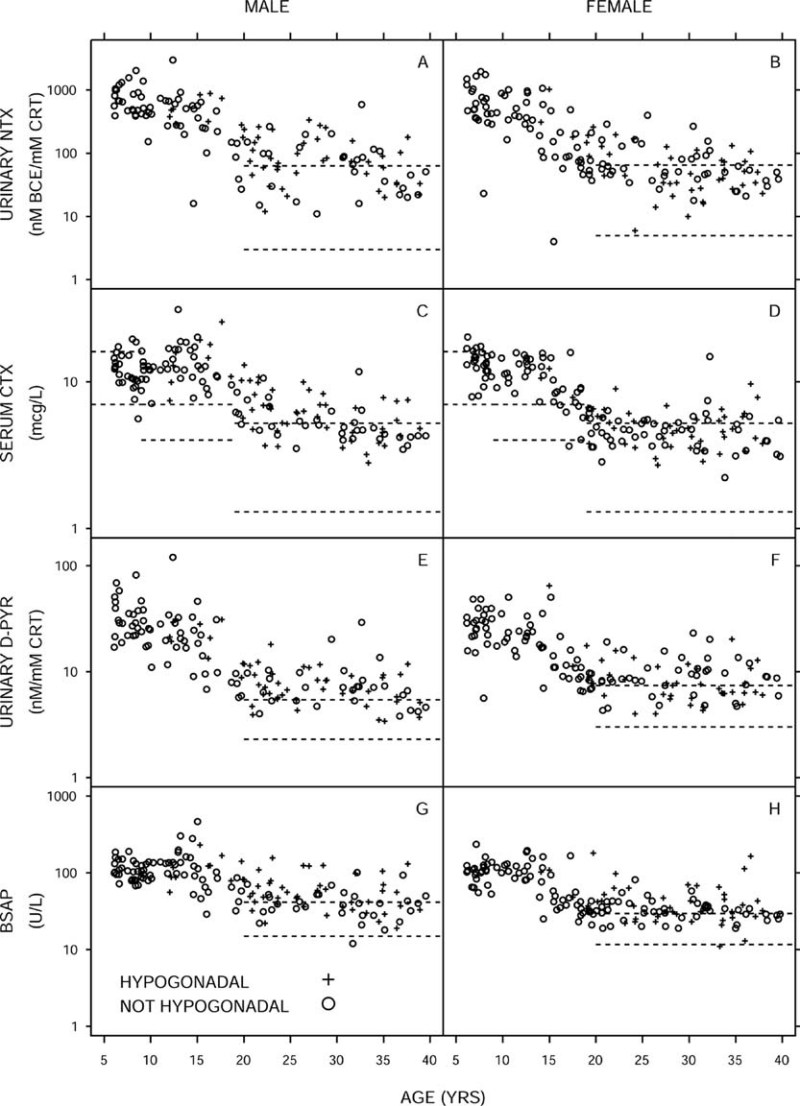
Bone turnover markers vs. age stratified by sex and hypogonadal status (hypogonadal, +; not hypogonadal, ○ ) with age-dependent upper and lower limits of normal (dashed lines). One individual age 75 yr is omitted.

Serum and urinary concentrations of bone turnover markers were plotted for all ages (Fig. 4), overlaying available normative data. Limited pediatric normative data are available. Bone turnover is high during childhood and adolescence compared with adults, reflecting rapid bone development. Adults, regardless of the presence or absence of hypogonadism, had substantially elevated levels of both bone resorption and formation markers, indicating elevated bone turnover.

### Self-reported fractures

By participant self-reporting and review of medical records, fractures occurred in 36% of participants, with 8.9% reporting three or more lifetime fractures (Table 2). Extremity fractures were most common at 33%, followed by back and hip fractures at 3.6%. The cumulative risk for fractures increased almost linearly with age (*p* < 0.001). Overall, no sex difference was seen; however, among participants <20 yr of age, males were more likely to fracture compared with females (mean ratio, 2.5; 95% CI, 1.1–5.6; *p* = 0.026). Asian participants reported lower fracture rates than whites (*p* = 0.025).

**FIG. 5:**
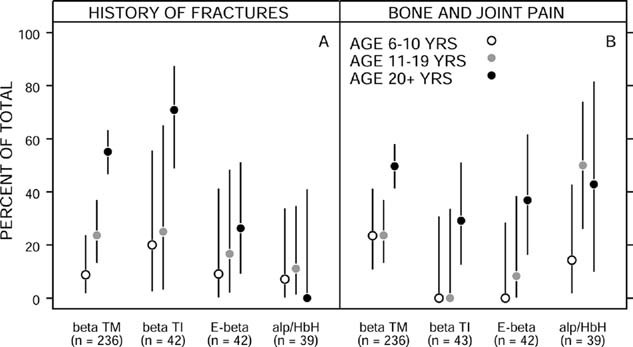
Prevalence of history of fracture (A) and recent bone or joint pain (B) by thalassemia syndrome, stratified by age group (6–10 yr, ○; 11–19 yr, 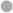; 20 or more yrs, •). Point estimates and exact 95% confidence intervals are indicated.

The fracture incidence rate varied significantly among the thalassemia syndromes (*p* = 0.014), with E-β and α thalassemia participants having lower fractures rates compared with β TM participants (mean ratio [95% C1]: E-β, 0.40 [0.20,0.81]; HbH/CS, 0.10 [0.01,0.70]). The prevalence of a history of fractures among adults was 55% in TM and 71% in TI (Fig. 5); this difference was not significant. The effect of thalassemia syndrome was lost in multivariate analysis.

Spine and femur BMD Z-score and total body BMC were negatively associated with fracture rate. For a 1-SD decrease in spine or femur BMD Z-score, the mean fracture rate increased by 37% or 47%, respectively (*p* < 0.001 for both). After controlling for age, higher D-Pyr concentrations, hypogonadism, histories of medicated heart disease, and hepatitis C were associated with higher fracture rates. Other bone turnover markers, other endocrinopathies, transfusion and chelation histories, and childhood activity were not significantly associated with fracture rate. In a multiple regression model selected by stepwise regression, increased fracture rates were independently associated with lower spine BMD Z-score, greater relative weight, and history of hepatitis C (Table 4).

### Bone and joint pain

Thirty-four percent of participants reported having bone or joint pain during the 30 days before enrollment (Fig. 5). Six percent required prescription pain medications and an additional 12.2% used over-the-counter analgesics (Table 2). Age, sex, and thalassemia syndrome were all independent predictors of the presence and severity of bone and joint pain (Table 4). The odds of more severe pain increased 47% for each 5-yr age increment. Forty percent of females but only 28% of males complained of recent pain. Bone pain was reported more frequently among β TM participants (40%) compared with β TI and E-β participants (16% and 19%, respectively) (Fig. 5). Reported bone and joint pain was not associated with bone mass or fractures. Age at starting transfusion or chelation, units of blood transfused, and ferritin and transferrin receptor concentrations did not correlate with the presence or severity of bone pain, indicating that iron overload and transfusion-related parameters did not explain the higher frequency of bone pain among TM participants. Participants with GH deficiency reported more severe bone pain (OR, 2.33; 95% CI, 1.11–4.76; *p* = 0.022), as did those with histories of medicated heart disease, cirrhosis, or hepatitis C, but these were not independent of age, sex, and thalassemia syndrome. Hypogonadism, other endocrinopathies, serum vitamin D concentrations, total calcium intake, and weight and height measurements were not associated with bone pain. Participants who reported being more active at the time of the study reported less pain, but this likely reflects the fact that people in pain are usually less active, not the converse.

### Vertebral morphometry

#### Vertebral fractures

MXA was available in a total of 352 participants. Nine participants (2.6%) had evidence of vertebral fractures by radiographic review, whereas only seven participants (2.0%) reported a history of vertebral fracture by interview. All vertebrae with radiographic evidence of fracture had Genant compression deformity grades 2 or 3. Eight of the nine participants with vertebral fractures were β TM and one was β TI. The mean age was 48.6 ± 11.5 yr, with the youngest being 37 yr of age. Vertebral fracture prevalence increased with age (OR for 5-yr increase: 2.54; 95% CI, 1.74–4.29; *p* < 0.001). After controlling for age, spine BMD Z-scores were negatively associated with fractures (OR for 1-SD increase: 0.32; 95% CI, 0.12–0.71; *p* = 0.01). Neither sex, hypertransfusion regimen, years or age at onset of DFO therapy, serum transferrin receptor or ferritin concentration, endocrinopathies, or anthropometric parameters correlated with fractures. Seven participants with vertebral fractures were hypogonadal (78%), seven reported back pain (78%), and all nine reported recent bone and joint pain.

#### Vertebral growth disturbance

Thirty-one participants (9.0%) had vertebral growth disturbances (VGDs) defined as a uniformly decreased height of several vertebrae. Prevalence of these abnormalities did not differ significantly by age, sex, or race. The mean age was 24.3 ± 4.3 yr (range, 18–34 yr). Twenty-nine (94%) of the participants with VGD were β-TM. Among participants with β TM, initiation of DFO therapy after age 6 yr (OR, 0.22; 95% CI, 0.10–0.45; *p* < 0.001), higher spine BMD Z-scores (OR for 1-SD increase, 0.57; 95% CI, 0.37–0.86; *p* = 0.009), and increased urinary D-Pyr (OR for 2-fold increase, 0.15; 95% CI, 0.05–0.40; *p* < 0.001) were all independently associated with lower odds of VGD (Table 4). Whereas not an independent predictor of VGD, hypogonadism was positively associated (OR, 3.44; 95% CI, 1.50–8.39; *p* = 0.004) after adjusting only for age. Presence of VGD was not specifically correlated with either generalized bone pain, joint pain, or back pain.

Eighteen (5.2%) participants evaluated for vertebral morphometry had abnormalities that involved individual vertebrae (e.g., presence of ring apophysis, platyspondyly, Schmorl's nodes; Table 2), 17 of whom were β TM. A total of 45 participants (12.8%) had either VGD or isolated vertebral abnormalities.

## DISCUSSION

This study found a high prevalence of low BMD, fractures, and bone pain in both β and α thalassemia syndromes. It showed a strong association between bone mass and fractures, and it identified factors that may contribute to the pathogenesis of bone disease in thalassemia. Bone disease would be expected to be less severe with mild or moderate hemolysis, as is generally found in patients with β TI, HbH disease, or HbH/CS. Indeed, bone mass was lower among β TM and E-β and higher among β TI, HbH disease, and HbH/CS. However, the effect of thalassemia syndrome seemed to be caused by underlying differences in growth, hypogonadism, and bone turnover, so that hypogonadal and lighter patients with high bone turnover had a higher likelihood of low BMD among all thalassemia syndromes.

Bone mass was reduced even in children-only 45% of 6- to 10-yr-old participants had normal BMD. We found a strong negative association between bone mass and age in the 11- to 19-yr age group. Peak bone mass was suboptimal. Short stature in some patients may have influenced these results, which are based on areal BMD measurements. However, the mean height Z-score was close to the population mean, and correlations with volumetric BMD did not suggest an important effect of bone size on our findings. Lower weight and higher bone turnover were the only two independent predictors of bone mass in patients <20 yr of age, whereas hypogonadism, transfusion and chelation parameters, and family history of osteoporosis were not found to play roles. These data indicate that adolescence is a critical period for the development of low bone mass in thalassemia and suggest that bone turnover plays a central role in this process. Similar to other reports,(23,24) we found increased bone turnover in adults with thalassemia, regardless of hypogonadism, and our limited pediatric normative data suggest that the same is true during adolescence. Altogether, it is possible that the increased bone turnover during this time does not allow for positive bone accrual and attainment of optimal peak bone mass. Neither the etiology of increased bone turnover in thalassemia nor a possible disassociation between bone formation and resorption can be addressed in this study. One may hypothesize, however, that the increased resorption in thalassemia relates to a state of increased oxidative stress,(25) which has been shown to be present in these patients, and which can also result in increased TNF production and bone loss.(26,27) Because current chelation and transfusion regimens have remained largely unchanged in this cohort over the last 20 yr, it seems that current thalassemia therapies do not prevent or retard the development of low bone mass.

Hypogonadism was a strong independent predictor of low bone mass. Surprisingly, we did not find higher BMD among hypogonadal subjects who received gonadal steroid replacement compared with those untreated. It is possible that this is related to the cross-sectional design of the study, the delayed or inadequate replacement with gonadal steroids, or intermittent compliance with the prescribed treatment. Gonadal steroid replacement schemes in females of this study were frequently similar to the regimens used in postmenopausal women, which may be inadequate for optimal bone accrual.(28) Nonetheless, the potential beneficial effect of gonadal steroid replacement in thalassemia should not be dismissed based on this study. An improvement of BMD in thalassemia with gonadal steroid replacement has been reported in some studies but not others.(11),(29–31) Thus, this subject needs to be rigorously evaluated by longitudinal randomized studies testing various types of gonadal replacement. Further longitudinal studies are also needed to determine the optimal serum 25 vit D concentrations for normal bone mass accrual and maintenance in thalassemia,(32,33) because we found high rates of vitamin D deficiency and insufficiency, as well as a negative and nonlinear relationship between spine BMD and 25 vit D concentrations. These findings may have significant clinical implications because both screening and therapy of vitamin D deficiency are easy, affordable, and beneficial. Information about fluctuations in 25 vit D concentrations over the course of years and their effect on the bone is not available in this study. Overall, the cross-sectional design of this study constitutes a limiting factor in our ability to infer the etiology of bone disease in thalassemia. This limitation, as well as sample size considerations, may also explain the lack of association between bisphosphonate treatment and bone mass. In addition, because there was no control group, our data were compared with standard databases, and the results should be interpreted with this in mind.

Years on regular transfusions, DFO treatment, and transfusion rates were used to assess the long-term effects of thalassemia management and iron overload on the bone. No association was found. Current degree of iron overload (assessed by serum ferritin concentration) was also not associated with bone disease. It is possible that the role of iron in the pathogenesis of the disease was missed because we lacked a definitive marker of iron overload. Ferritin is a poor marker in heavily overloaded individuals. Other measures of iron excess, for example, liver biopsy, superconductive quantum interference device (SQUID), or MRI, were not performed in this study. Finally, ineffective erythropoiesis (assessed by serum transferrin receptor concentrations) was not associated with bone mass, with the exception of an association with femur BMD in univariate analysis of unclear biological significance.

The fracture prevalence based on both patient history and medical record review was 36%. This is substantially lower than the rates of 50% reported in 1960s and 1970s.(34) Our present results differ from a recent TCRN report that observed an overall fracture prevalence of 12%.(35) The latter report(35) most likely underestimated the fracture prevalence because of its reliance on chart review of a TCRN established database that was generic in nature and not comprehensive for prior bone problems. In this study, E-β and α thalassemias were found to have lower rates of fractures compared with β TM. However, as in the case of our BMD results, neither thalassemia syndrome nor chelation and transfusion parameters were significant independent predictors of fracture rates in multivariate analysis. Instead, a strong association between BMD and fracture rate was observed. We also found that, in these patients, as in osteoporotic patients, the cumulative risk for fractures increased with age in an almost linear fashion. A bimodal fracture distribution with one peak related to sports activities among adolescent males and a second rise related to osteoporosis among postmenopausal females has been described in the general population.(36–38) Such a bimodal fracture risk was not observed among our patients. Similar to the previous retrospective TCRN study,(35) children and adolescents did not seem to be at increased risk for fractures. This finding may be related to the cross-sectional design of our study or to the decreased level of physical activity in this patient population.

Whereas long bone metaphyseal irregularities and abnormal vertebral bodies resembling bone dysplasias have been described in β TM and attributed to DFO toxicity,(39) the presence and etiology of these abnormalities given today's standard of care are largely unknown. This is the first study in thalassemia to use vertebral morphometry to evaluate a large number of patients for such abnormalities. Indeed, a large number of deformities were found, mostly VGDs characterized by decreased height of multiple vertebrae. VGD occurred exclusively in regularly transfused and chelated patients with β TM and E-β, were associated with early use of DFO (before age 6), and occurred regardless of age, with the youngest affected patient being 18 yr old. A rather small number of vertebral fractures were documented, found primarily in patients with β TM. Vertebral fractures increased with age and were related to low bone mass and hypogonadism. These data suggest different etiologies between vertebral fractures and deformities, and in particular, the role of early treatment with DFO in the development of vertebral deformities in thalassemia.

Despite its high prevalence, the etiology of bone pain in our patients remains obscure. There was no correlation with history of fractures or vertebral deformities, except in those with documented vertebral fractures by morphometry, who almost always reported bone pain. Bone pain increased with age and was more frequent among females, despite fractures rates similar to those of males. Patients with β TM also reported bone pain more frequently than those with other thalassemia syndromes, although we were unable to establish any transfusion or chelation related parameter as a possible contributor to pain. Neither endocrinopathy, other thalassemia complications, nor vitamin D deficiency were independent predictors of bone pain. Why females with β TM are more prone to bone pain remains uncertain.

In summary, in this large cohort of patients across all thalassemia syndromes, we observed a high prevalence of fractures and a strong association between bone mass and fractures. Therefore, strategies to improve BMD are very important in thalassemia management. We showed that bone disease in thalassemia is an adolescent problem with adult manifestations. Current transfusion and chelation practices seem insufficient to prevent the development of low bone mass. Our data highlight the need for randomized trials to determine the appropriate form of gonadal steroid replacement and vitamin D supplementation as well as additional strategies to optimize bone accrual in this disease. Further longitudinal studies are needed to address changes of bone mass during puberty. Finally, changes in bone turnover seem to be involved in the development of bone disease, although the factors that lead to increased bone resorption in thalassemia remain unclear and warrant further study.

## Appendix 1

This is Publication 6 of the Thalassemia Clinical Research Network (TCRN).This work was performed through the TCRN. The authors thank the patients who volunteered their time to participate in this study. The following TCRN sites and investigators also contributed to the study (listed in alphabetical order): Childrens Hospital, Boston: Ellis Neufeld, MD, PhD, Principal Investigator, Melody Cunningham, MD, Co-Principal Investigator; Childrens Hospital of Philadelphia: Alan R Cohen, MD, Principal Investigator, Janet L Kwiatkowski, MD, Co-Principal Investigator, Catherine S Manno, MD, Coinvestigator, Marie Martin, RN, Nurse Coordinator, Debra Hillman, Regulatory Affairs Coordinator, Gail M Jackson, CDT, Nutrition Assessment Program Coordinator, Maria J Henwood-Storto, DO, Endocrinologist; Shannon H Fourtner, MD, Endocrinologist; Childrens Hospital & Research Center Oakland: Elliott Vichinsky, MD, Principal Investigator, Dru Foote, NP, Study Coordinator, Eun-Ha Pang, Study Coordinator, Zahra Pakbaz, MD, CCD, Certified Clinical Densitometrist, Selma Holden, Study Coordinator; Toronto General Hospital: Nancy Olivieri, MD, Principal Investigator; U.T. Southwestern Medical Center: Charles T Quinn, MD, Assistant Professor of Pediatrics, Elizabeth Shull, BSN, RN, Clinical Research Associate; Weill Medical College of Cornell University: Patricia J Giardina, MD, Principal Investigator, Robert W Grady, PhD, Co-Investigator, Jeffrey E Mait and Dorothy Kleinert, NP, MPH, MA, Study Coordinators, Irina Chaikhoutdinov and Gladys Cintron, Data Coordinators, Sylvia Hom, DXA Technician, Hospital for Special Surgery; National Heart, Lung, and Blood Institute: Charles Peterson, MD, Project Officer; Data Coordinating Center, New England Research Institutes: Sonja McKinlay, PhD, Principal Investigator, Felicia Trachtenberg, PhD, Statistician, Haddy Jallow, MS, MPH, Project Coordinator.
